# Echocardiographic evaluation of changes in diastolic dysfunction after CABG surgery, effect of novel therapy with Ivabradine

**DOI:** 10.1186/1749-8090-8-S1-O295

**Published:** 2013-09-11

**Authors:** D Panayotova, M Slavov, Y Krasnaliev, PL Panayotov

**Affiliations:** 1 University Hospital St. Marina, Varna, Bulgaria

## Background

The majority of patients undergoing surgical revascularization for multi-vessel coronary artery disease have some degree of left ventricular diastolic dysfunction. The purpose of the study is to evaluate the therapeutic effect of Ivabradine added to the medication of such patients early after CABG surgery.

## Methods

Two groups of patients with triple-vessel coronary disease were evaluated. Each group consisted of 15 patients,aged between 40 and 70, 70% being males and 30% females.The patients in both groups had similar systolic left ventricular function and some degree of diastolic dysfunction. Group I patients had standard postoperative medical treatment, and in Group II supplemental therapy with Ivabradine 5 – 7.5 mg bid was added early after surgery. Patients with concomitant valvular and/or pericardial disease were excluded. The echocardiography was performed with M-mode and Two-dimensional examination, Pulsed wave Doppler imaging and Tissue Doppler imaging. The following echocadiographic criteria for diastolic dysfunction evaluation were used: LA dimension mm; LA Volum mL/m²; E/A; E m/s and A m/s; DTE ms; e’ cm/s and a’ cm/s; E/e’; Ar – A dur ms (Ar=duration of pulm. venous A-wave, Adur=duration of mitral inflow (Ar=duration of pulm. venous A-wave, Adur=duration of mitral inflow A-wave); IVRT ms; S/D. Patients were examined before, 7 days and 3 months after surgery.

## Results

We observed a tendency towards normalization of e’ wave, and E/e’ in Group II. Reduced Ar-Adur was found as well. Changes in DTE and LA volume were another tendency, found in Group II.Such tendencies are not observed in Group I. Significant changes of variables within the Ivabradine treated group, reaching the normal range, are shown in graphics.

## Conclusion

Ivabradin added to the standard postoperative medical therapy probably contributes to improved diastolic function in post-CABG patients. Prospective data from bigger groups are necessary to obtain more significant conclusions.

